# Chronic nonbacterial osteomyelitis in neuroradiology – behavior and evolution of vertebral and mandibular lesions on imaging

**DOI:** 10.1007/s00247-024-06079-0

**Published:** 2024-10-25

**Authors:** José Sá Silva, Sofia Bettencourt, Inês Madureira, Marta Conde, Carla Conceição

**Affiliations:** 1 Centro Hospitalar Universitário de Santo António, Unidade Local de Saúde de Santo António, Rua Prof. Vicente José de Carvalho 37, 4050-366 Porto, Portugal; 2 Hospital Dona Estefânia, Unidade Local de Saúde de São José, Lisbon, Portugal

**Keywords:** Chronic osteomyelitis, Chronic recurrent multifocal osteomyelitis, Computed tomography, Magnetic resonance imaging, Mandible, Pediatric, Spinal column, Vertebral body

## Abstract

**Background:**

Chronic nonbacterial osteomyelitis (CNO) is a rare non-infectious inflammatory musculoskeletal disease where imaging plays a key diagnostic role. Vertebral and mandibular lesions are frequent manifestations, meaning their awareness is crucial for the neuroradiologist to avoid delays in diagnosis and treatment.

**Objective:**

Characterize vertebral and mandibular CNO lesions on imaging to assist practicing neuroradiologists in better identifying this disease.

**Materials and methods:**

Retrospective review of all CNO patients of our pediatric center, including only patients with vertebral or mandibular lesions. All imaging exams were analyzed to record lesion characteristics.

**Results:**

We included 13 patients (six male). The mean age of onset was 12.3 years. Ten patients had only vertebral lesions, two had only mandibular lesions, and one had both. For patients with vertebral lesions, the median number of levels affected was three, 81.8% had multiple levels affected, 90.0% had dorsal spine lesions, 72.7% had platyspondyly, and 81.8% had inflammatory changes. All vertebral lesions had at least partial resolution of inflammatory findings, the mean time of lesion activity was 2.5 years, and recurrence occurred in 27.3%. Three patients had sacral lesions, all with sacroiliitis. In patients with mandibular lesions, all had unilateral lesions involving the mandibular ramus, all had hyperostosis, periosteal reaction, bone edema, and soft tissue inflammation, all had partial resolution on follow-up, and one had recurrence.

**Conclusion:**

CNO vertebral lesions are not rare, are often multiple, predominantly affect dorsal levels, and most result in vertebral height loss. Resolution of vertebral inflammatory lesions is frequent, but so is recurrence. Sacral lesions may be present and result in sacroiliitis. The mandible may be a site of unifocal disease, typically affecting the ramus, with prominent bony changes and soft tissue inflammation.

**Graphical Abstract:**

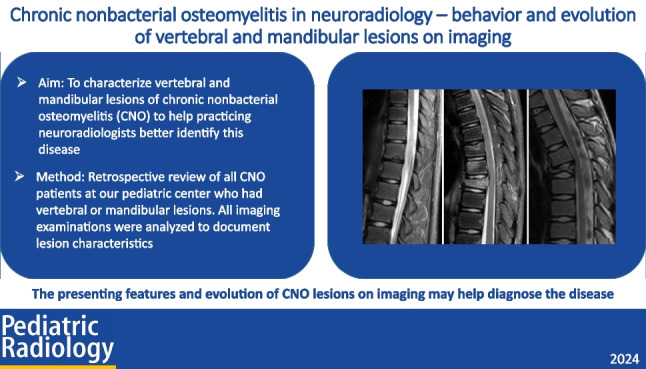

## Introduction

Chronic nonbacterial osteomyelitis (CNO), first described in 1972 by Giedion et al. [1], represents an inflammatory condition affecting the musculoskeletal system, manifesting across a broad clinical spectrum. The term chronic recurrent multifocal osteomyelitis (CRMO) has also been used in the literature to describe this disorder, but since recurrency or multifocality is not a mandatory feature [2, 3], the term CNO might be more accurate.

While the exact cause of CNO pathogenesis remains unclear, sterile microbiological analysis and the uselessness of antibiotic treatment disfavor an infectious cause [4]. It has been hypothesized that CNO might have an important genetic component [5].

Although rare, its incidence is probably underestimated, and increasing cases have been reported [6]. Low awareness of the disease often leads to considerable delays between the onset of symptoms and diagnosis [3, 7].

Clinical presentation is heterogeneous and the disease usually presents with an early teenage onset. Pain is the most prominent and frequent feature in CNO. Most patients develop insidious bone pain, with nocturnal pain being reported in a subset of patients. Physical exam might be normal, but focal tenderness, warmth, and swelling may be present. Systemic symptoms can also be present, such as low-grade fever, poor growth, and weight loss. The course of the disease can be continuous, recurrent, or continuous and recurrent [3, 8].

Multifocal involvement and cutaneous features may be present, and some authors considered CNO to be a pediatric form of the adult synovitis, acne, pustulosis, hyperostosis, and osteitis (SAPHO) syndrome [9, 10], although the term SAPHO should only be used for patients with cutaneous manifestations to avoid confusion.

CNO lesions most frequently affect the metaphysis of long bones, especially within the lower extremities. The pelvis and clavicles are also often involved [2, 8]. Vertebral lesions are variably reported in the literature, ranging from 8 to 38% [2, 11, 12]. Mandibular involvement reports range from 2 to 21% [2, 13]. Clinically silent bone lesions can occur and may affect vertebral bodies, which is relevant given that the most common complications of CNO include platyspondyly and vertebral pathological fractures, which may be present in about 10% of patients at the time of diagnosis [8, 11]. The number of lesions at initial onset is quite variable, and if unifocal lesions are present, the clavicle and mandible are most likely affected [2, 3].

Magnetic resonance imaging (MRI) is the most sensitive imaging modality for CNO lesions, permitting a better visualization of the inflammatory changes in bony structures and surrounding soft tissues, which are the most typical findings of CNO. MRI can also detect sequelae of past CNO lesions, like bone marrow adipose substitution or sclerosis. For these reasons, whole-body MRI is considered the gold standard for imaging, providing information on the distribution of lesions [3, 8]. Classic radiographs can be used as a fast initial screening method, detecting not only bone sclerosis and cortical thickening, but also other abnormalities in more severe cases, like sequestrum (a piece of de-vascularized bone that becomes separated from the remainder of the bone in osteomyelitis), bone destruction, and cortical erosion [14]. For the spine, radiographs can be particularly useful in screening for vertebral body lesions with height loss. Despite this, classic radiographs may not detect early inflammatory changes, potentially delaying diagnosis. Bone scintigraphy shows abnormal tracer uptake in active and recovering lesions, even if clinically silent [15]. Although this could be useful for screening clinically silent lesions where MRI is unavailable, scintigraphy requires radiation exposure and is less sensitive than MRI [16]. Computed tomography (CT) also involves radiation exposure but may be complementary to MRI for better characterizing bony lesions, and better depicting mixed lytic and sclerotic changes, periosteal reaction, bony expansion, and possible fractures [17].

Laboratory test results are usually non-specific, with elevated C-reactive protein (CRP) and erythrocyte sedimentation rate (ESR) frequently present. Because there are no specific disease markers available, no laboratory tests can be considered reliable for monitoring disease activity [2, 3, 8].

Although bone biopsy of the lesions is often performed, no specific histopathologic patterns have been reported in the literature, so its role is mainly to exclude differential diagnoses [3, 18]. Even though the exclusion of other etiologies may be crucial, especially when we consider CNO to be a diagnosis of exclusion, we must note that performing a biopsy is an invasive procedure, with associated risks. Clinical data and radiological findings may obviate the need for biopsy in some patients [19], but it may still be needed when clinical presentation and imaging findings are inconclusive.

Treatment strategies are variable, with nonsteroidal anti-inflammatory drugs (NSAIDs) commonly used as a first-line treatment, while secondary treatment options include glucocorticoids, sulfasalazine, methotrexate, tumor necrosis factor-alpha (TNF-α) inhibitors, bisphosphonates, and anti-interleukin-1 receptor agents. Treatment of CNO with NSAIDs or disease-modifying agents is favorable in most patients [3, 20].

The reported recurrence and remission rates of the disease vary greatly, with higher recurrence rates being reported in series where patients had longer follow-up periods. Favorable outcomes may be related to earlier and sufficient treatment [2, 8, 21].

Head, neck, and spine imaging exams are frequently reported by neuroradiologists who, in our understanding, might have a low awareness of this disease. This might be because, before a clinical diagnosis is made, mandibular and spinal CNO lesions may be seen as unspecific and thus misdiagnosed. Also, whole-body MRI is usually requested only after a clinical diagnosis of CNO is established and is usually reported by non-neuroradiologists, which reduces exposure of the disease to neuroradiologists. Because of this, and because spinal lesions are associated with poorer outcomes [22], we believe it is important to raise awareness to avoid unnecessary delays in diagnosis and treatment. Therefore, the objective of this study is to find and characterize the imaging features of vertebral and mandibular lesions of CNO, to raise awareness and to assist practicing neuroradiologists in better identifying this disease.

## Methods

### Patients and clinical data

The medical records of all CNO patients followed in our center with disease onset before 18 years of age were retrospectively reviewed by a pediatric rheumatologist and a neuroradiologist (M.C., with 23 years of experience, and J.S.S., with 4 years of experience, respectively). Diagnoses were made by local physicians and were based on the presence of uni- or multifocal bone inflammatory lesions, persistent or recurrent symptoms, and the exclusion of differential diagnoses. In reviewing the medical records, we retrospectively confirmed that the patients met the Bristol diagnosis criteria [19]. We then selected only the patients that had vertebral or mandibular lesions, or both.

Data were collected on the following clinical characteristics: sex, age at symptom onset, time from symptom onset to diagnosis, follow-up time, symptoms during disease course, and treatment approach.

Laboratory data of inflammatory markers was collected, namely, CRP level, and ESR.

Histological findings, when available, were also registered.

All patient data have been completely anonymized throughout the data collection and writing of the manuscript and related files.

### Imaging

All imaging studies available at our picture archiving and communication system (PACS) were reviewed in consensus by two of the authors (C.C., a pediatric neuroradiologist with 28 years of experience, and J.S.S.). We obtained data on the number and local of lesions, lesion patterns, and follow-up intervals. Lesion patterns were based on the classification used by Andronikou et al. [23] and we classified patients into appendiculo-axial pattern, claviculo-spinal pauci-focal pattern, and unifocal or only axial pattern.

We then specifically assessed vertebral and mandibular lesions to define lesion characteristics.

Regarding patients with vertebral lesions, we recorded the following: the number of vertebral lesions and the affected vertebral segments; the presence and severity of platyspondyly, dividing severity in three groups—more than 50% of vertebral body height loss, less than 50% of height loss, and Schmorl nodes (Schmorl nodes refer to protrusions of the intervertebral disc through the vertebral body endplate, and where combined with platyspondyly for simplification); the presence and severity of active inflammatory changes on any MRI exam, dividing inflammatory severity into two groups – only bone edema, and soft tissue inflammation (defined as increased signal intensity within perilesional soft tissues adjacent to the bony cortex); resolution of inflammatory findings on follow-up imaging exams, dividing patients into three groups – absent resolution, partial resolution (inflammatory findings severity decreased over time, but still present on the last follow-up exam), and total resolution (inflammatory findings completely absent on last follow-up exam); time of lesion activity, defined as the number of years during which inflammatory changes could be detected on follow-up exams; recurrence of inflammatory lesions on imaging; the presence of sacroiliac joint inflammatory changes; the presence of costovertebral joint involvement; the presence of vertebral endplate involvement; the presence of posterior vertebral elements involvement; the presence of intervertebral disc lesions; the pattern of vertebral edema, divided into focal or diffuse; the presence of chronic changes to the normal spinal curvature.

Regarding patients with mandibular lesions, we evaluated the following: the affected sites of the mandible; the presence of hyperostosis (referring to excessive growth or thickening of bone tissue); the presence of active inflammatory changes (bone edema and soft tissue inflammation); the presence of periosteal reaction; resolution of inflammatory findings on follow-up imaging exams (categorized as absent, partial, or total resolution); recurrence of inflammatory lesions on imaging.

### Statistical analysis

The data collected in this study was analyzed using SPSS Statistics (version 27; IBM Corp., Armonk, NY). Descriptive statistics, including mean, standard deviation (SD), median, and interquartile range (IQR), were calculated for continuous variables, as appropriate, while frequencies were calculated for categorical variables. The normality of data distribution was examined using skewness and kurtosis.

## Results

### Demographics and clinical characteristics

The total number of patients with CNO followed in our center, with disease onset before 18 years of age, was 29. We excluded all patients with no vertebral or mandibular lesions and included the remaining 13.

Of the included 13 patients, six were male (46%). The mean age at symptom onset was 12.3 years (± 2.4; range 7–16). The mean time from symptom onset to diagnosis was 15.2 months (± 14.2; range 1–51). The mean time of follow-up was 5.0 years (± 2.7; range 1.5–11.0). Regarding symptoms presented during the disease course, all the patients with vertebral lesions complained of back pain, and only 18% had accompanying fever; all the patients with mandibular lesions had local pain and tumefaction, with no fever. Table 1 summarizes patient characteristics.

**Table 1:** Patient characteristics (*n* = 13)

Sex – *n* (%)			
Male	6	(46.2)	
Female	7	(53.8)	
Age at symptom onset – years, mean ± SD	12.3	±2.4	(range 7–16)
Time from symptom onset to diagnosis – months, mean ± SD	15.2	±14.2	(range 1–51)
Follow-up time – years, mean ± SD	5.0	±2.7	(range 2–11)
Symptoms – *n* (%)			
Patients with vertebral lesions (*n*=11)			
Back pain	11	(100.0)	
Fever	2	(18.2)	
Patients with mandibular lesions (*n*=3)			
Local pain and tumefaction	3	(100.0)	
Fever	0	(0.0)	
Elevated inflammatory markers – *n* (%)			
CRP	8	(61.5)	
ESR	11	(84.6)	
Level of inflammatory markers – mean ± SD			
CRP (mg/L)	16.5	±14.9	
ESR (mm/h)	34.3	±16.4	
Total number of lesions – median (IQR)	3	(4)	(range 1–18)
Patients by lesion location – *n* (%)			
Patients with vertebral lesions	11	(84.6)	
Patients with mandibular lesions	3	(27.3)	

*CRP* C-reactive protein, *ESR* erythrocyte sedimentation rate, *IQR* interquartile range, *SD* standard deviation

The first-line therapy for all patients consisted of NSAIDs, usually followed by corticosteroids (in 62% of all patients), then followed by more aggressive treatment such as bisphosphonates (in 62% of patients), classic disease-modifying anti-rheumatic drugs (DMRADs), anti-TNF-α agents, and non-anti-TNF-α Biological DMRADs.

Regarding inflammatory markers, CRP level was increased in 62% of patients with a mean value of 16.5 mg/L (± 14.9), and ESR was increased in 85% of patients with a mean value of 34.3 mm/h (± 16.4) (Table 1).

Biopsy was performed in seven patients (54%), with two showing features consistent with chronic osteomyelitis, four showing nonspecific findings, and one being inconclusive.

### Imaging

The median total number of lesions on imaging exams was 3 (IQR 4; range 1–18). As to the lesion distribution patterns, 31% of the patients had an appendiculo-axial pattern, 15% had a claviculo-spinal pauci-focal pattern, and 54% had a unifocal or only axial pattern.

Regarding the anatomical distribution of lesions, of the 13 patients included, 10 had vertebral lesions without mandibular lesions, two had mandibular lesions with no vertebral lesions, and one had vertebral and mandibular lesions. A more thorough analysis of this distribution is presented in the following sections.

Follow-up intervals had a median time of 12 months (IQR 9.5; range 1–66).

#### Imaging – vertebral lesions

**Fig. 1:**
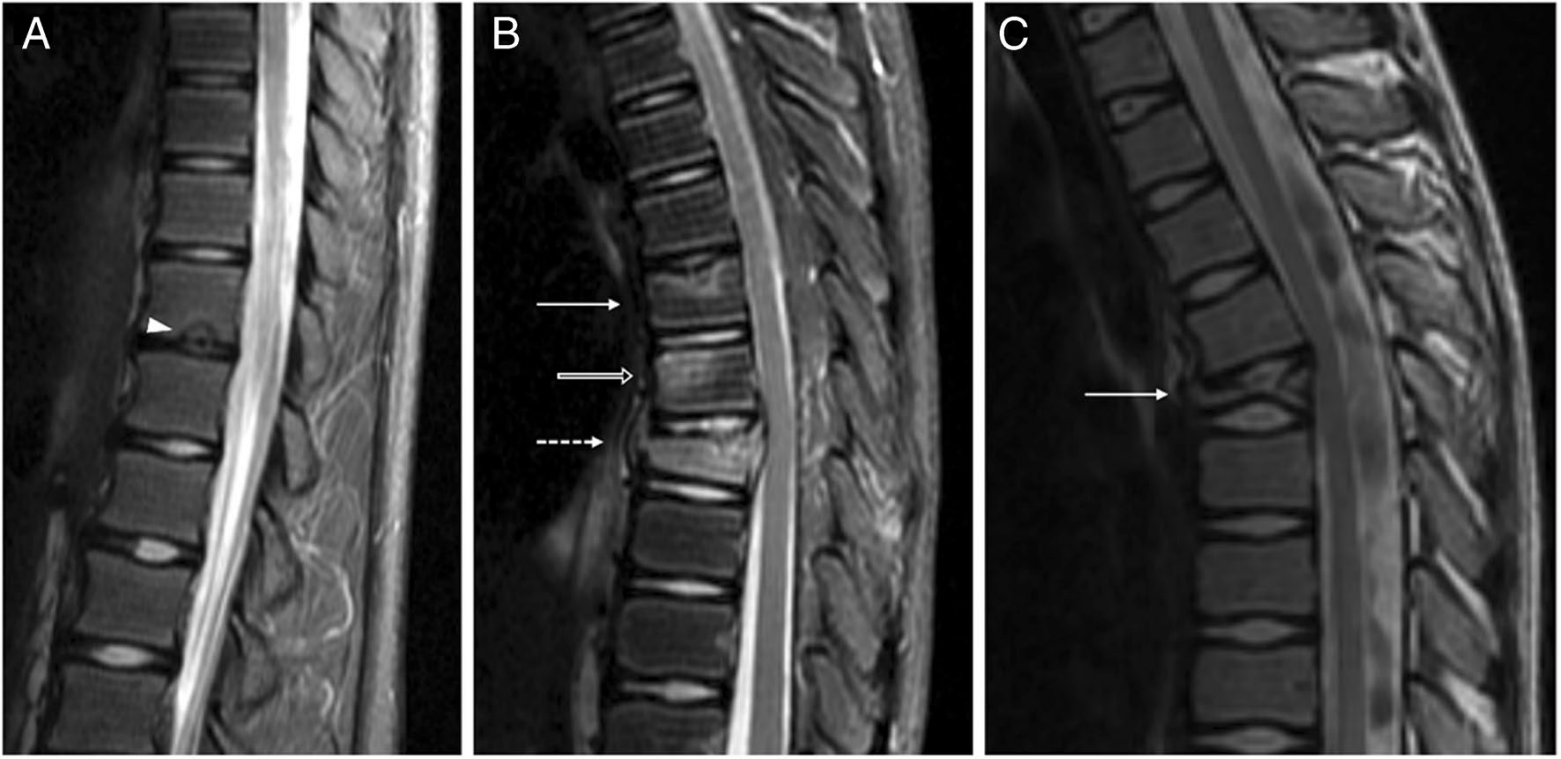
Schmorl node and platyspondyly with different degrees of vertebral height loss in three different patients with chronic nonbacterial osteomyelitis: magnetic resonance images of the dorsal spine obtained from a 14-year-old boy (**a**), a 12-year-old boy (**b**), and a 12-year-old girl (**c**). **a** Sagittal T2-weighted fat-saturated image shows a Schmorl node in the inferior endplate of dorsal (D) vertebra 11 (*arrowhead*). **b** Sagittal T2-weighted fat-saturated image shows platyspondyly of D6 with less than 50% of vertebral body height loss (*arrow*), bone edema of the body of D7 (*open arrow*) without vertebral height loss, and platyspondyly of D8 with more than 50% of vertebral body height loss (*broken arrow*). Note also the bone edema adjacent to the superior endplate in D6, and the pre- and post-somatic soft tissue inflammation around D8. **c** Sagittal T2-weighted image shows severe vertebral height loss of D6, with a vertebra plana appearance (*arrow*)

Of the total 13 patients, 11 had vertebral lesions. Patients with vertebral lesions had a median number of vertebral levels affected of 3 (IQR 2; range 1–9). Nine patients (82%) had multiple vertebral levels affected, and two (18%) had only one level affected. In relation to the vertebral segments affected, 18% of the patients had cervical lesions, 91% had dorsal lesions, 27% had lumbar lesions, and 27% had sacral lesions. All patients with vertebral lesions had dorsal involvement, except for one patient whose vertebral lesions were only sacral. Eight patients (73%) had platyspondyly, with two (18%) having lesions with more than 50% of vertebral body height loss, four (37%) having lesions with less than 50% of vertebral body height loss, and two (18%) having only Schmorl nodes (Fig. 1).

**Fig. 2:**
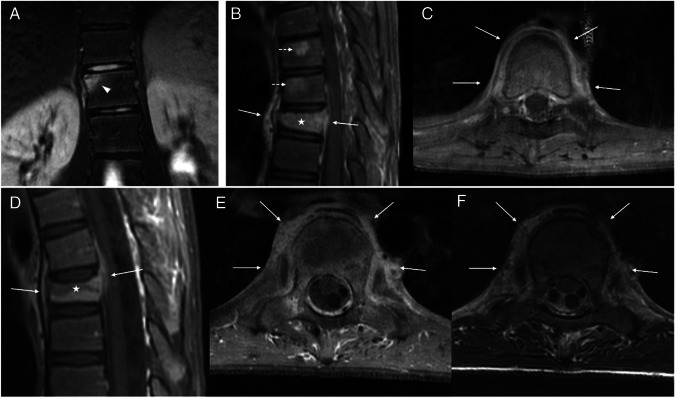
Types of inflammatory changes in vertebral lesions: magnetic resonance images obtained from a 16-year-old girl (**a**), a 12-year-old boy (**b**, **c**), and a 14-year-old boy (**d**–**f**). **a** Coronal T2-weighted fat-saturated image of the dorsolumbar spine shows a lesion of the body of dorsal (D) vertebra 12 with only bone edema (*arrowhead*). **b**,** c** Sagittal (**b**) and axial (**c**) T1-weighted contrast-enhanced fat-saturated images of the dorsal spine reveal vertebral height loss and contrast enhancement of the body of D8 (*asterisk*), and the presence of enhancing soft tissue inflammation with a peri-vertebral distribution (*arrows*). Note also the enhancing inflammatory lesions of the D6 and D7 vertebral bodies (*broken arrows*). **d**–**f** Sagittal (**d**) and axial (**e**) T1-weighted contrast-enhanced fat-saturated images and axial T2-weighted (**f**) image of the dorsal spine show platyspondyly of D6 (*asterisk*) and extensive peri-vertebral soft tissue inflammation (*arrows*). Note the involvement of the costovertebral joints

Nine patients (82%) had active inflammatory changes on imaging, with four (36%) showing only bone edema, and five (45%) showing (in addition to bone edema) soft tissue inflammation (Fig. 2). Soft tissue inflammation had a para-vertebral distribution in four patients and a pre-sacral distribution in one. In five patients, we observed, with the resolution of inflammatory findings during the course of the disease, transformation of bone edema into fatty deposits.

None of the patients with platyspondyly or soft tissue inflammation had spinal cord compression.

**Fig. 3:**
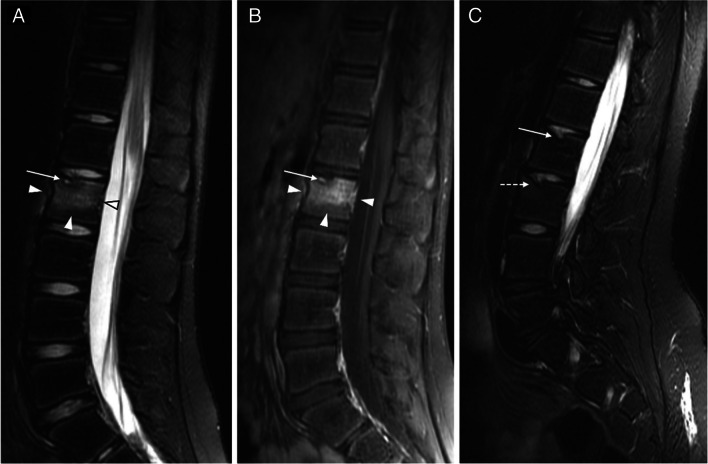
Resolution of inflammatory findings in vertebral lesions: magnetic resonance images of the lumbar spine obtained from a girl at ages 12 (**a**, **b**) and 16 (**c**) years. **a**,** b** Sagittal T2-weighted fat-saturated (**a**) and sagittal T1-weighted contrast-enhanced fat-saturated (**b**) images of the lumbar spine show bone edema and enhancement of the body of lumbar (L) vertebra 2 (*arrowheads*). There is also a Schmorl node of the superior vertebral endplate of L2 (*arrow*). **c** Sagittal T2-weighted fat-saturated image of the same patient 4 years later shows the complete resolution of the inflammatory changes of L2, with resolution of the edema. There is a persistent Schmorl node of the superior vertebral endplate of L2 (*arrow*), representing a permanent sequela. Note also another Schmorl node in L3 (*broken arrow*), representing a sequela of another lesion that occurred in the time between the two exams

**Fig. 4:**
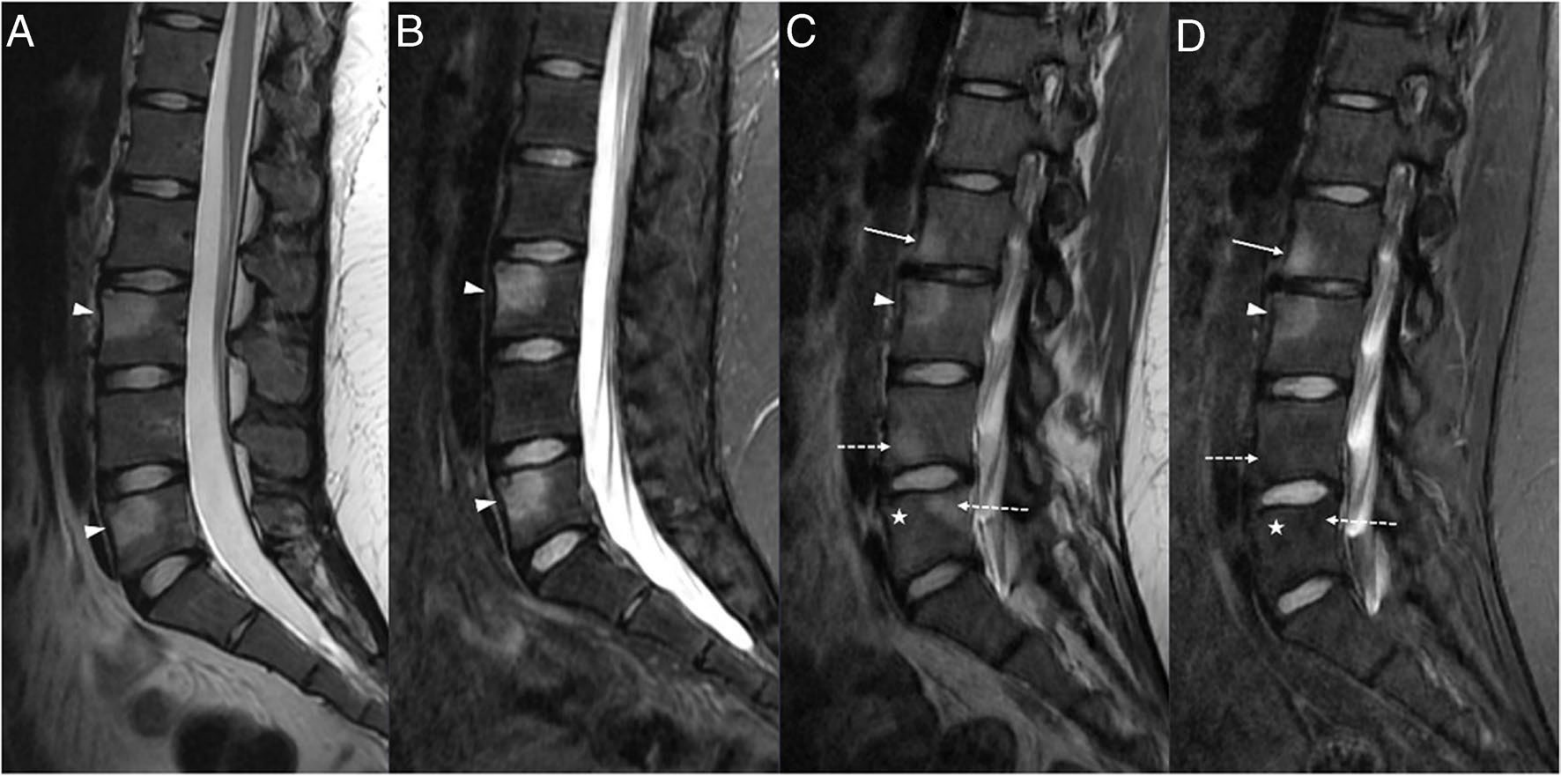
Evolution of vertebral lesions and recurrence: magnetic resonance images of the lumbar spine obtained from a girl at ages 15 (**a**, **b**) and 17 (**c**, **d**) years. **a**,** b** Sagittal T2-weighted (**a**) and sagittal T2-weighted fat-saturated (**b**) images show bone edema in the bodies of lumbar (L) vertebra 3 and L5 (*arrowheads*). **c**,** d** Sagittal T2-weighted (**c**) and sagittal T2-weighted fat-saturated (**d**) images of the same patient about 2 years later show a new inflammatory lesion with bone edema in L2 (*arrow*), as well as partial resolution of the previous L3 lesion (*arrowhead*), complete resolution of the previous L5 lesion (*asterisk*), and areas of fat replacement in L4 and L5 representing sequelae of resolved inflammatory lesions that appeared in the time between the two exams (*broken arrows*)

All patients had resolution of inflammatory findings on follow-up imaging exams, with five (45%) showing total resolution, and six (55%) showing partial resolution (Figs. 3 and 4). On average, patients with partial resolution had more anatomical sites affected on imaging, with a mean of 7.2 affected sites (SD ± 5.8) compared with a mean of 3.0 (SD ± 0.7) in patients with total resolution. For patients with total resolution, the mean time of lesion activity was 2.5 years (SD ± 1.3; range 1.5–4).

Four patients (36%) had chronic changes of the normal spinal curvature occurring as a consequence of vertebral body height loss with wedge deformity. All four patients had accentuation of the dorsal kyphosis, and one had also a concomitant dorsal scoliosis.

Vertebral endplate involvement occurred in eight patients (73%), represented by irregularity or depression of the endplate, always occurring in levels with platyspondyly. Isolated involvement of posterior vertebral elements occurred in only one patient, who had multiple vertebral levels affected with lesions of the pars interarticularis and the articular processes. Four other patients had edema of the pedicles originating from the extension of vertebral body lesions. Disc lesion occurred in six patients (55%), in all cases with disc height loss, with or without loss of the normal disc T2 hyperintensity, and always occurring in levels with adjacent platyspondyly.

Regarding the pattern of vertebral bone edema, of the nine patients with edema, four had only diffuse edema of the vertebral bodies, and four had simultaneously vertebral levels with diffuse involvement and levels with only focal edema. The one patient who had isolated involvement of posterior vertebral elements showed only focal edema.

Recurrence of vertebral inflammatory lesions on follow-up imaging was observed in three (27%) patients, and it always occurred in vertebral levels not previously affected (Fig. 4). The mean time to recurrence was 2.1 years (SD ± 1.7; range 0.8–4).

**Fig. 5:**
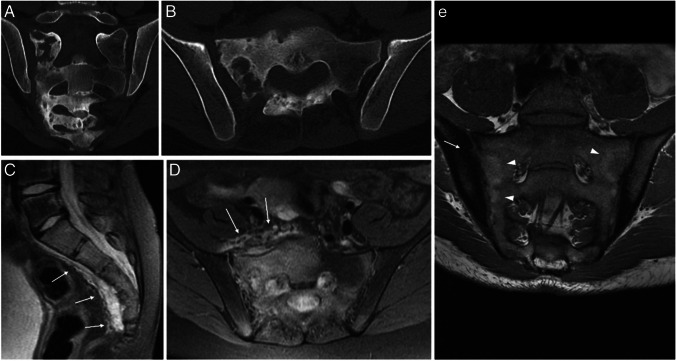
Sacral involvement in two patients with chronic nonbacterial osteomyelitis: images of the sacrum obtained from an 11-year-old girl (**a**–**d**) and a 17-year-old boy (**e**). **a**,** b** Coronal (**a**) and axial (**b**) computed tomography images of sacral inflammatory lesions, where severe changes of the bone architecture can be seen, with mixed lytic and sclerotic changes, cortical destruction, and some thickening of the bony structures. **c**,** d** Sagittal (**c**) and axial (**d**) proton density weighted fat-saturated magnetic resonance (MR) images of the same patient reveal extensive sacral bone edema and pre-sacral soft-tissue inflammation (*arrows*). **e** Coronal T1-weighted MR image of another patient in a chronic non-active phase shows sequelae of past inflammatory lesions, with fat substitution on the sacral sides of the sacroiliac joints (*arrowheads*) and sclerosis on the iliac sides, with articular surface irregularities (*arrow*)

Of the three patients that had sacral lesions, all had signs of sacroiliitis and subchondral sclerosis, two of them showed sacroiliac synovitis, and two had bilateral involvement (Fig. 5). Three patients (27%) had involvement of costovertebral joints, always at levels where there were vertebral lesions. In Tables 2 and 3, there is a summary of the characteristics of the patients with spine involvement and the characteristics of vertebral lesions.

**Table 2:** Characteristics of patients with vertebral lesions (*n* = 11)

**Total vertebral levels affected** – median (IQR)	3	(2)	(range 1–9)
**Vertebral levels affected** – *n* (%)			
Multiple	9	(81.8)	
Single	2	(18.2)	
**Vertebral segments affected** – *n* (%)			
Cervical	2	(18.2)	
Dorsal	10	(90.9)	
Lumbar	3	(27.3)	
Sacral	3	(27.3)	
**Platyspondyly** – *n* (%)	8	(72.7)	
≥ 50% of vertebral body height loss	2	(18.2)	
< 50% of vertebral body height loss	4	(36.4)	
Schmorl nodes	2	(18.2)	
**Active inflammatory changes on imaging** – *n* (%)	9	(81.8)	
Soft tissue inflammation	5	(45.5)	
Only bone edema	4	(36.4)	
**Resolution of inflammatory findings on follow-up imaging exams**—*n* (%)			
Total	5	(45.5)	
Partial	6	(54.5)	
Absent	0	(0.0)	
**Time of lesion activity** – years, mean ± SD			
For totally resolved lesions	2.5	± 1.3	
**Recurrence of vertebral inflammatory lesions on follow-up imaging** – *n* (%)			
Levels not previously affected	3	(27.3)	
Levels previously affected	0	(0.0)	
**Time until recurrence** – years, mean ± SD	2.1	± 1.7	
**Sacral lesions** – *n* (%)	3	(27.3)	
Sacroiliitis	3	(27.3)	
Subchondral sclerosis	3	(27.3)	
Sacroiliac synovitis	2	(18.2)	
Bilateral involvement	2	(18.2)	

*IQR* interquartile range, *SD* standard deviation

**Table 3:** Individual patient data for vertebral lesions (*n* = 11)

Patient number	Number of vertebral lesions	Affected segments	Platy-spondyly	Active inflammatory changes	Resolution of inflammatory findings	Recurrence of inflammatory lesions	Time of lesion activity, years	Sacroiliitis	Costo-vertebral joint involvement
1	1	D	PP	BE	Partial	No	4.0	No	No
2	2	D	PP	N	Total	No	NA	No	No
3	3	D	N	N	Total	No	NA	No	Yes
4	2	S	N	BE	Partial	No	1.6	Yes	No
5	9	C, D, L, S	PP	BE, STI	Partial	Yes	3	Yes	Yes
6	6	D, L	PP	BE	Partial	No	0.5	No	No
7	3	D, L	PP	BE, STI	Total	Yes	4	No	No
8	2	D, S	PP	BE, STI	Partial	No	1	Yes	No
9	3	D	TP	BE, STI	Total	No	1.5	No	No
10	2	D	TP	BE, STI	Total	Yes	2	No	Yes
11	4	C, D	N	BE	Partial	No	1	No	No

*BE* only bone edema, *C* cervical, *D* dorsal, *L* lumbar, *N* none, *NA* not applicable, *PP* partial platyspondyly with less than 50% of vertebral body height loss, *S* sacral, *SN* Schmorl node, *STI* soft tissue inflammation, *TP* total platyspondyly with more than 50% of vertebral body height loss

#### Imaging – mandibular lesions

**Fig. 6:**
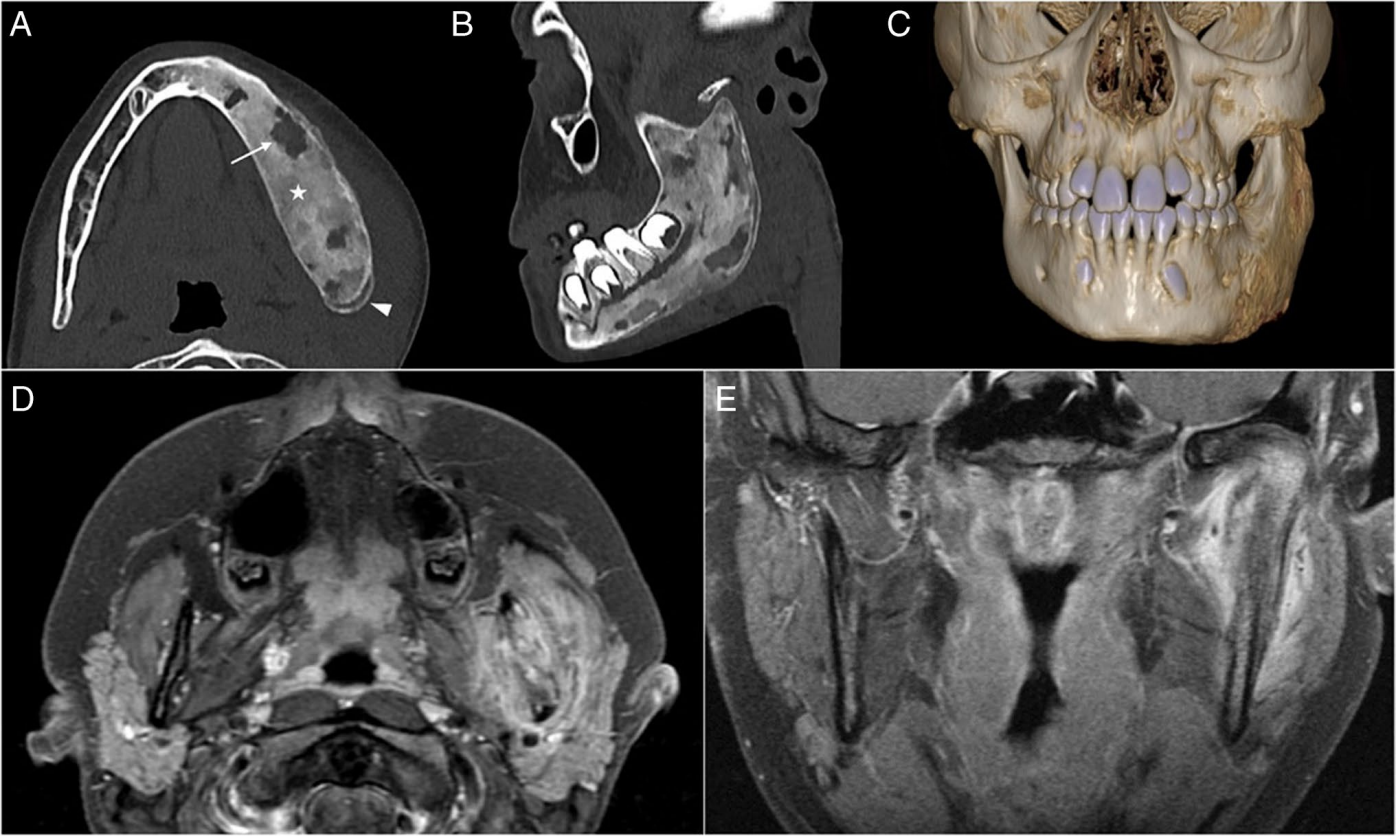
Imaging characteristics of mandibular lesions on computed tomography (CT) and magnetic resonance (MR): images obtained from an 8-year-old girl (**a**–**c**) and from a 14-year-old girl (**d**, **e**). **a**–**c** Axial (**a**), coronal (**b**), and three-dimensional reconstructed (**c**) CT images of the mandible reveal an extensive lesion of the left mandibular body and ramus, with bone expansion, heterogeneous ground-glass density (*asterisk*), lytic areas (*arrow*), cortical irregularity, and periosteal reaction (*arrowhead*). **d**,** e** Axial (**d**) and coronal (**e**) T1-weighted contrast-enhanced fat-saturated MR images of another patient show severe inflammatory changes of the soft tissues surrounding the left mandibular ramus

Of the total 13 patients, there were three with mandibular lesions. One of these patients also had vertebral involvement, but the other two had unifocal disease of the mandible. In all patients, the mandibular involvement was unilateral, and the affected mandibular sites were the angle and the ramus (including condylar and coronal processes), with two patients also having the ipsilateral body of the mandible involved. All patients showed hyperostosis, periosteal reaction, bone edema, and soft tissue inflammation. Two patients had CT scans performed that showed mixed density bone lesions with osteolytic foci (Fig. 6).

**Fig. 7:**
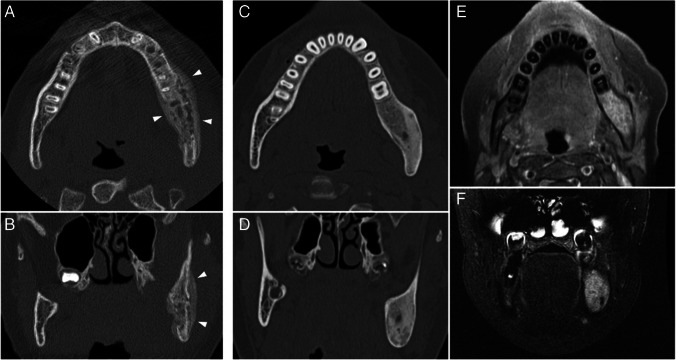
Evolution of mandibular lesions: images obtained from a boy at ages 12 (**a**, **b**) and 13 (**c**–**f**) years. **a**,** b** Axial (**a**) and coronal (**b**) computed tomography (CT) images of the mandible show an active inflammatory process in the left body and ramus, with a heterogeneous pattern, predominantly lytic, with cortical erosion, and with periosteal reaction (*arrowheads*). **c**,** d** Axial (**c**) and coronal (**d**) CT images of the same patient 1 year later show the inflammatory process has partially resolved, and that the bone is now expanded but conserves its cortical borders, showing a more diffuse ground-glass density. **e**,** f** Axial T1-weighted contrast-enhanced fat-saturated (**e**) and T2-weighted fat-saturated (**f**) magnetic resonance images reveal there are still signs of inflammation, with contrast enhancement and bone edema seen on (**e**) and (**f**), respectively

All patients had partial resolution of the mandibular lesions on follow-up imaging exams (Fig. 7). Only one patient had recurrence, with recrudescence of inflammatory changes at the same site of the previous lesion, after 11 years.

## Discussion

Our investigation describes in detail the imaging findings expected in vertebral and mandibular lesions of CNO and their evolution during the course of the disease.

The demographic characteristics of the patients in this study do not differ much from other published studies. More females than males were affected, with symptom onset usually during early adolescence. Our time until diagnosis was somewhat longer than other studies, which may reflect still some lack of awareness of this entity [2, 3, 17, 24]. Back pain was a significant clinical marker of vertebral disease, as already previously reported [17, 24]. The total number of vertebral bone lesions per patient was also similar to a previous report [24].

Follow-up intervals were quite variable between our patients since our center did not follow a specific rule to time out follow-up exams, and rather based the execution of imaging exams on clinical aggravation.

In patients with spine lesions, involvement of multiple vertebral levels was the most common presentation, with dorsal levels predominantly affected. Vertebral body height loss was an important complication, showing up in more than half of our patients and leading to spinal curvature deformities in some cases. Resolution of vertebral inflammatory lesions, total or partial, occurred in all patients, but recurrence appeared in about a third. Sacral lesions, when present, always affected the sacroiliac joints.

Several studies showed that vertebral involvement is frequent [3, 11, 12, 24–26], ranging up to 38% in the general population [12] and to 81% in a pediatric population [26], suggesting vertebral involvement might be higher in children. In our sample, 45% of the patients with vertebral lesions had no other anatomical locations affected, suggesting that limited involvement of the spine in CNO might also be frequent. Similar to other studies, spine lesions most frequently affected the thoracic spine [12, 24, 26]. In our study, 82% of the patients had multiple vertebral levels affected. Another study described multifocal vertebral involvement in 60% of patients in a pediatric population [26]. These results suggest multifocality as a common finding of spine CNO lesions in children.

Most of our patients (55%) had lesions with vertebral height loss. A previous large study described vertebral fractures in 17.5% of the patients with spine involvement [2]. Considering that fractures usually result in vertebral height loss, this value is low when compared to ours. However, our results are more similar to those of other studies where spinal lesions were specifically evaluated with MRI, which found vertebral body deformities in 52% and vertebral height loss in 64% of the patients with spine involvement [24, 26]. The patients in our study all performed MRI, which is more sensitive than other imaging modalities and may explain these different results. We also showed that vertebral lesions in children can lead to abnormal kyphosis and scoliosis, with 36% of our patients having spinal deformities, similar to what has been previously reported [12, 26].

Vertebral endplate involvement and disc lesions occurred in 73% and 55% of our patients, respectively, differing from a study by Guariento et al., where disc involvement was found in 26% and endplate abnormality in 57% [26], but these values show that endplate and disc involvement may be frequent regardless. In our study, these abnormalities almost always occurred adjacent to vertebrae with platyspondyly, a tendency that Guariento et al. also recognized. Finally, although the vertebral body is the region that is classically injured in CNO, with possible extension to the pedicles, we had one patient who showed isolated involvement of the posterior vertebral elements in several levels. This pattern was also described in one patient by Guariento et al., highlighting that, although rare, it should be recognized.

Active inflammatory changes on imaging appeared in almost all our patients, except two, in which only sequelae of previous vertebral lesions were found. All patients with active inflammation had bone edema, and almost half had surrounding soft tissue inflammation with a para-vertebral or pre-sacral distribution. These findings on MRI are not specific of CNO and rather represent an inflammatory process, but are similar to other descriptions of CNO lesions, vertebral or otherwise [17, 27, 28].

Total or partial resolution of vertebral inflammatory lesions on follow-up imaging was present in all our patients. Treatment approaches across our patients were variable, and due to a small sample size we could not establish a relationship between the treatment used and the degree of lesion resolution. However, all our patients were treated, and our results acknowledge the response of vertebral lesions to therapy, but the spontaneous resolution of spine lesions could not be evaluated. The best treatment strategy for CNO is yet to be completely determined. NSAIDs have been shown to be an effective first treatment option [29]. Consensus treatment plans for CNO refractory to NSAIDs have been established [30], but the comparison of effectiveness between different treatments is still lacking [31]. Other studies proposed bisphosphonates as an effective treatment option for CNO patients with vertebral lesions, with good results [21, 24]. One study demonstrated partial or complete resolution of vertebral MRI hyperintensities in every patient treated with pamidronate [24]. This emphasizes how imaging may be an important mean to monitor the evolution of lesions and treatment response. As such, whole-body MRI has been proposed as the best imaging modality to monitor disease activity, but the ideal follow-up intervals have not been clearly established [28, 29, 32].

Recurrence rates of CNO have been variably reported, from as low as 16% to as high as 83%, and may vary according to follow-up time and treatment used [13, 21, 33, 34]. In our study, the recurrence rate of vertebral lesions on MRI was inside this interval, but recurrence rates on imaging may be lower than clinical recurrence of symptoms, since some of our patients had recurrent symptoms not always accompanied by imaging findings. We also found that all patients with recurrent vertebral lesions on MRI had its recurrence at vertebral levels not previously affected, and that recurrence can appear as late as after 4 years of follow-up.

Reported sacral involvement is about 19% in CNO patients with vertebral lesions [24, 26], which is similar to what we found, but varies from 24 to 72% in all CNO patients [17, 35]. Sacroiliitis has been reported in 72% of CNO patients, with most having bilateral involvement, similar to our results [35]. A previous study showed that unilateral sacroiliac involvement is more frequent when only clinical evaluation is performed, but bilateral involvement is more frequent when imaging is used [36]. This again underlies the importance of sensitive imaging studies in the investigation of CNO. d’Angelo et al. described sacroiliac involvement as affecting one (iliac or sacral) or both sides of the sacroiliac joint, or the sacroiliac synovial space [17], findings that have also been observed in our study.

CNO is a diagnosis of exclusion, and its imaging appearance can be non-specific, so the differential diagnosis of vertebral lesions and platyspondyly in the pediatric age should always be considered. Infectious osteomyelitis, which can be very hard to differentiate from CNO solely on imaging, should be excluded based on clinical features and other complementary diagnostic tests, sometimes requiring biopsy. Depending on the epidemiologic context, tuberculous and other granulomatous infectious diseases should be suspected. Langerhans cell histiocytosis is the most common cause of vertebra plana (loss of almost the entire vertebral body height) in children and can have soft tissue involvement, underscoring its inclusion in the differential. However, Langerhans cell histiocytosis may have other differentiating features on imaging that are not typical of CNO, and biopsy can be diagnostic. Lymphoproliferative disorders can infiltrate vertebral bones and sometimes resemble osteomyelitis, but, unlike CNO lesions, tend to progress along adjacent vertebral levels with a more infiltrative pattern. Bone metastasis can present heterogeneously, possibly resembling osteomyelitis, but are rare in this age group and should be considered mainly if there is a known primary tumor.

Our study demonstrated that the mandible can be a site of unifocal disease, typically affecting its ramus, with prominent bony changes and soft tissue inflammation. These results mirror those of the current literature. The mandible is recognized as a classic site of involvement in CNO [2, 13, 17, 28] and is a known site of unifocal disease [13]. Chronic osteomyelitis usually affects one hemimandible, involving the ascending ramus and the condylar process [37, 38]. Prevalence of mandibular lesions varies from 2 to 21% [2, 13].

MRI is sensitive for early detection of bone and adjacent soft tissue inflammation in patients with mandibular osteomyelitis [37], but CT can also be useful since these patients usually have extensive bone changes [38, 39]. Previous reports describe these lesions as having mixed lytic and sclerotic abnormalities, diffusely distributed, with areas of resorption of medullary and cortical bone, sequestrum, and periosteal reaction. Inflammation of surrounding soft tissues is a common feature, best seen on MRI [37, 39]. The patients in our study mimic these descriptions.

Another important point is how these lesions evolve over time. When active inflammation subsides, the bone matrix becomes more sclerotic, with less pronounced lytic changes, with redefinition of bone contours, and reduction of contrast uptake; periosteal new bone apposition during acute phases may cause persistence of bone thickening, leading to mandibular enlargement [37]. This is a pattern we observed in our patients. Even though active inflammation improved, sequelae from the lesions persisted.

Imaging helps evaluate response to treatment. Bisphosphonates are described as a treatment option for mandibular chronic inflammatory lesions [38, 40, 41], but we could not evaluate the impact of treatment in mandibular lesions due to our small sample size.

The main limitations of this study are its small sample size and its retrospective nature, which hindered a more reliable evaluation of lesion patterns and relationships, and limited the generalizability of the results, possibly concealing rarer manifestations not included in our results. We cannot exclude selection biases as the patients treated in our pediatric referral hospital were probably referred due to severe manifestations, excluding milder cases from our analysis. Also, our clinicians based the execution of follow-up imaging on clinical aggravation, meaning that patients with milder clinical episodes may not have been imaged. A case–control prospective study could better evaluate the relationship between imaging and clinical features, and larger sample sizes and follow-up times could unravel features and imaging patterns not observed in our study.

In CNO, vertebral involvement is not rare, and neuroradiologists may encounter this disease during their work life, highlighting the importance of knowing it.

Vertebral lesions are often multiple, affecting predominantly dorsal levels, with most patients showing vertebral body height loss. Resolution of vertebral lesions, partial or total, is frequent, but recurrence is not uncommon. Sacral lesions are a feature of CNO, frequently affecting the sacroiliac joints. Mandibular lesions may be a site of unifocal disease, and the mandibular ramus is typically affected, with surrounding soft tissue inflammation.

For practicing neuroradiologists, a key feature for distinguishing CNO lesions is the involvement of multiple anatomical sites, especially if typical sites are involved, like the long bone metaphysis of the lower extremities, pelvis, clavicles, and spine. For isolated involvement of the spine, involvement of several levels with diffuse edema of the vertebral body, platyspondyly, and predilection for dorsal levels should raise suspicion. Bilateral involvement of the sacroiliac joints and adjacent bone also seems to be characteristic of CNO. Isolated mandible lesions represent a more challenging diagnosis, but lesions that only partially recover over time and recurrence at the same site may be suggestive.

Although the prognosis of CNO is considered good, with the resolution of symptoms in most patients, about a quarter can have persistent disease and sequelae [42, 43]. Studies with longer periods of follow-up may be necessary to investigate how CNO evolves over time and the factors that influence its progression. The ideal follow-up intervals for radiological monitoring of CNO patients are still not determined, and future longitudinal studies on the long-term outcomes of these patients could help clarify this question. Validating the imaging findings in a larger, more diverse population could also improve the generalizability of our results. Finally, exploring the underlying pathophysiological mechanisms leading to specific imaging findings could provide directions for future research.

## Data Availability

No datasets were generated or analysed during the current study.
